# Dominant Role of Polypropylene Chain Architecture in Differentiating Flame Retardancy and Mechanical Performance

**DOI:** 10.3390/polym18111356

**Published:** 2026-05-29

**Authors:** Shu Yin, Menghan Guo, Hao Wang, Lin Wang, Xiangmei Li, Jiyu He

**Affiliations:** 1 School of Material Science and Engineering, Beijing Institute of Technology, Beijing 100081, China; 2 National Engineering Research Center of Flame Retardant Materials, Beijing Institute of Technology, Beijing 100081, China

**Keywords:** polypropylene, intumescent flame retardancy, melt rheology, interfacial compatibility

## Abstract

Achieving a synergistic improvement in flame retardancy and mechanical performance remains a persistent challenge in intumescent flame-retardant (IFR) polypropylene (PP) systems. Previous studies have predominantly focused on optimizing flame retardant formulations while largely overlooking the critical role of polymer matrix chain architecture in determining the overall composite performance. In this work, three PP matrices with distinct chain architectures—homopolymer (hPP), random copolymer (rPP), and block copolymer (bPP)—were systematically investigated within an identical IFR formulation. The results reveal a dominant role of chain architecture in differentiating flame retardancy and mechanical performance, which are governed by distinct structural factors, namely melt rheological behavior and phase morphology. Specifically, bPP exhibits superior flame retardancy, as evidenced by a higher limiting oxygen index (LOI) and improved UL 94 rating, which may be associated with its higher melt viscosity and resistance to dripping during combustion. In contrast, rPP shows significantly improved mechanical performance, owing to its more homogeneous phase structure and enhanced chain mobility. These findings demonstrate that flame retardancy and mechanical properties can be effectively tuned through different structural pathways, providing a viable strategy to mitigate the conventional trade-off in IFR systems. This work highlights the importance of polymer chain architecture as a complementary design parameter alongside flame retardant additives for developing high-performance PP composites.

## 1. Introduction

Intumescent flame-retardant (IFR) systems have been widely employed to improve the fire safety of polypropylene (PP) due to their advantages of low toxicity and high efficiency [1,2,3,4]. However, achieving a simultaneous improvement in flame retardancy and mechanical performance remains a long-standing challenge, as the incorporation of IFR additives often deteriorates the mechanical properties of PP, leading to an inherent trade-off between fire safety and structural integrity [5,6,7].

A typical IFR system generally consists of an acid source, a carbon source, and a gas source. During combustion, the acid source promotes dehydration and carbonization of the polymer or charring agent, the carbon source contributes to the formation of a carbonaceous framework, and the gas source releases non-flammable gases that expand the char layer [8]. Ammonium polyphosphate (APP) is commonly used as an acid source, while triazine-based charring agents (CF) can enhance char formation [9,10,11]. Piperazine pyrophosphate (PAPP) has also attracted attention because it can provide acid, gas, and carbon sources simultaneously. Nevertheless, single-component or poorly matched IFR systems often produce porous, fragile, or discontinuous char layers, which limits their flame-retardant efficiency [12,13]. To overcome these limitations, extensive efforts have focused on additive design and formulation optimization, including microencapsulation, surface modification, and synergistic combinations with inorganic nanomaterials [14,15,16,17]. Although these strategies can improve flame retardancy or partially mitigate mechanical deterioration, they remain predominantly additive-centric and rely mainly on external modification of the IFR system [18,19,20,21,22]. In contrast, the intrinsic characteristics of the polymer matrix, particularly chain architecture, have received comparatively limited attention in regulating the overall performance of PP/IFR composites [23,24,25,26,27].

As a semi-crystalline polymer, PP can exhibit different chain architectures, including homopolymer PP (hPP), random copolymer PP (rPP), and block copolymer PP (bPP). These matrices differ in chain regularity, comonomer distribution, crystallization behavior, and phase morphology [28,29]. For example, hPP generally possesses higher chain regularity and crystallinity, rPP contains randomly distributed comonomer units that reduce crystallinity and increase chain mobility, whereas bPP typically contains multiphase structures associated with hard and soft segments. These structural variations are known to influence melt rheological behavior, crystallization, and mechanical properties [30,31,32]. However, their roles in governing flame retardancy and mechanical performance in IFR systems remain insufficiently understood, especially under controlled conditions where the flame retardant formulation is kept constant [33,34]. Flame retardancy and mechanical properties are often discussed as if they were governed by similar structural factors, leading to the commonly observed but insufficiently examined trade-off between fire safety and mechanical integrity [35,36]. Few studies have systematically distinguished the respective contributions of melt rheological behavior and phase morphology to flame retardancy and mechanical performance in PP-based IFR composites [37,38,39,40,41].

In this work, three PP matrices with distinct chain architectures, namely hPP, rPP, and bPP, were systematically investigated within an identical IFR formulation. By keeping the additive composition constant, the intrinsic influence of PP chain architecture on composite performance was isolated. The results reveal a dominant role of chain architecture in differentiating flame retardancy and mechanical performance, which are governed by distinct structural factors, namely melt rheological behavior and phase morphology. This study provides new insights into the structure–property relationships of IFR systems and highlights polymer chain architecture as a complementary design parameter for developing high-performance flame-retardant PP composites.

## 2. Experimental Section

### 2.1. Materials

Homopolymer polypropylene (hPP, T30S) was supplied by China Petroleum & Chemical Corporation Sinopec, Maoming Branch, (Maoming, China), with an isotactic index higher than 96%. Random copolymer polypropylene (rPP, M250E) was obtained from Sinopec Shanghai Petrochemical Company Limited (Shanghai, China), while block copolymer polypropylene (bPP, K8003) was provided by Sinopec Beijing Yanshan Petrochemical Company (Beijing, China). Piperazine pyrophosphate (PAPP, JNP-2) was purchased from Sichuan Institute of Fine Chemical Industry Research and Design Co., Ltd. (Zigong, China). Ammonium polyphosphate (APP, JLS-APP101R) was supplied by Hangzhou JLS Flame Retardants Chemical Co., Ltd. (Hangzhou, China), and the charring agent (CF) was obtained from Qingyuan Riguang Dongcheng Chemical Co., Ltd. (Qingyuan, China).

Pentaerythritol tetrakis[β-(3,5-di-tert-butyl-4-hydroxyphenyl)propionate] (Irganox 1010, purity ≥ 94%) and tris(2,4-di-tert-butylphenyl) phosphite (Irgafos 168, purity ≥ 98%) were purchased from Macklin Biochemical Co., Ltd. (Shanghai, China), at loadings of 0.1 wt% (Irganox 1010) and 0.2 wt% (Irgafos 168), respectively.

### 2.2. Preparation of Composites

Three polypropylene matrices with different chain architectures were used in this study. The neat polymer is denoted as hPP (homopolymer PP), rPP (random copolymer PP), and bPP (block copolymer PP). When compounded with the intumescent flame-retardant (IFR) system, the corresponding composites are referred to as PP-1, PP-2, and PP-3, respectively. This nomenclature is applied consistently in all tables, figures, and text throughout the manuscript.

All samples were prepared in duplicate to ensure reproducibility. PP pellets were compounded with flame-retardant fillers at a total loading of 25 wt% using a twin-screw extruder at 170–185 °C and a screw speed of 10–15 rpm, followed by pelletization. The obtained pellets were dried in an oven at 100 °C for 8 h and subsequently injection-molded at 185–195 °C under a pressure of 90 MPa to produce standard specimens. Pure PP samples were fabricated under identical processing conditions. The detailed formulations are listed in Table 1.

### 2.3. Characterization Methods

The limiting oxygen index (LOI) was measured using an FTT007 oxygen index tester (Fire Testing Technology Ltd., East Grinstead, UK) in accordance with ASTM D2863-08 [42]. Specimens with dimensions of 125 mm × 6.5 mm × 3.2 mm were tested, and 5–10 samples were evaluated for each formulation.

The UL 94 vertical burning test was conducted following the ANSI/UL 94 standard [43] using a CZF-5A apparatus (Motis Fire Technology (China) Co., Ltd., Kunshan, China). Specimens with dimensions of 130 mm × 13 mm × 3.2 mm (or 1.6 mm) were employed. The flammability ratings were classified as V-0, V-1, V-2, or no rating (NR), with five parallel specimens tested for each sample.

Thermogravimetric analysis (TGA) was performed on a Netzsch 209F1 thermogravimetric analyzer (NETZSCH-Gerätebau GmbH, Selb, Germany) under a nitrogen atmosphere (flow rate: 50 mL/min). Approximately 5 mg of sample was heated from 40 °C to 800 °C at a heating rate of 10 °C/min. Each test was conducted in duplicate.

The melt flow index (MFI) was determined in accordance with ASTM D1238-23 [44] at 230 °C under a load of 2.16 kg. Each test was conducted in triplicate.

Cone calorimetry (CONE) tests were carried out using an FTT cone calorimeter (Fire Testing Technology Ltd., East Grinstead, UK) according to ISO 5660-1 [45]. Specimens (100 mm × 100 mm × 3 mm) were exposed to an external heat flux of 35 kW/m^2^. Each test was conducted in duplicate.

Dynamic rheological measurements were performed on a Haake MARS 40 rotational rheometer (Thermo Fisher Scientific, Karlsruhe, Germany) equipped with parallel plates (25 mm diameter) under a nitrogen atmosphere at 190 °C. Strain sweep tests (frequency: 1 Hz, strain range: 0.1–100%) were first conducted to determine the linear viscoelastic region. Frequency sweep tests were then carried out within the linear viscoelastic region (selected strain: 1%) over an angular frequency range of 0.1–100 rad/s to obtain the storage modulus (G′), loss modulus (G″), complex viscosity (|η*|), and loss factor (tan δ).

The microstructures of char residues after combustion and cryo-fractured surfaces were observed using a field-emission scanning electron microscope (FE-SEM, ZEISS GeminiSEM 300, Carl Zeiss Microscopy GmbH, Jena, Germany).

Raman spectroscopy was conducted using an HRM EVO Raman spectrometer (HORIBA France SAS, Palaiseau, France) with a laser wavelength of 532 nm to evaluate the graphitization degree of char residues. The spectral range was 500–2000 cm^−1^.

Thermogravimetric–infrared (TG-IR) analysis was carried out under a nitrogen atmosphere. Samples were heated from 40 °C to 800 °C at a rate of 10 °C/min, and the evolved gases were analyzed in real time using a Nicolet 6700 FTIR spectrometer (NETZSCH-Gerätebau GmbH, Selb, Germany) with a resolution of 4 cm^−1^.

Mechanical properties were evaluated using a universal testing machine (CMT-4104, Shenzhen SANS Testing Machine Co., Ltd., Shenzhen, China) according to ASTM D412-2006 [46]. Dumbbell-shaped specimens with a gauge length of 50 mm were tested at a crosshead speed of 50 mm/min. 6–10 samples were evaluated for each formulation.

Notched Izod impact strength was measured using a pendulum impact tester in accordance with ISO 180:2019 [47], employing V-notched specimens, and 6–10 samples were evaluated for each formulation.

Differential scanning calorimetry (DSC) was performed on a Netzsch 204F1 calorimeter (NETZSCH-Gerätebau GmbH, Selb, Germany) under a nitrogen atmosphere. Approximately 5 mg of sample was subjected to heating and cooling cycles from −30 °C to 200 °C at a rate of 10 °C/min. Each test was conducted in duplicate.

## 3. Results and Discussion

### 3.1. Thermal Stability and Flammability

**Figure 1:**
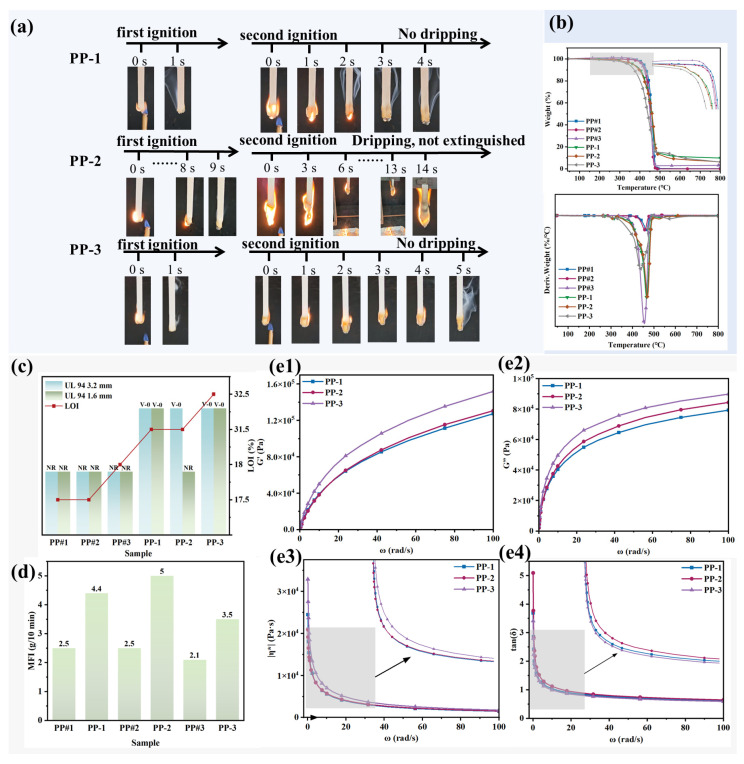
(**a**) Representative screenshots from the 1.6 mm vertical burning test; (**b**) TGA and DTG curves of the samples; (**c**) test results of LOI and UL 94; (**d**) melting index of PP/IFR samples; (**e1**–**e4**) frequency (ω) dependence of (**e1**) storage modulus (G′), (**e2**) loss modulus (G″), (**e3**) complex viscosity (|η*|) and (**e4**) loss tangent (tanδ) at for PP/IFR samples.

The flame retardancy of the PP/IFR composites was evaluated by limiting oxygen index (LOI) and UL 94 vertical burning tests, and the results are summarized in Figure 1c and Table S1. The neat PP is highly flammable and exhibits intense burning accompanied by severe melt dripping during the UL 94 test, resulting in no rating. Upon incorporation of IFR, the LOI values of all composites increase significantly, confirming the effective flame-retardant action of the system [48]. However, despite the comparable LOI values, distinct differences are observed in the UL 94 performance among the three composites. Specifically, the block copolymer-based sample (PP-3) achieves a UL 94 V-0 rating at both thicknesses without dripping, whereas the random copolymer-based sample (PP-2) fails at 1.6 mm due to burning to the clamp accompanied by severe melt dripping. The homopolymer-based sample (PP-1) also attains a V-0 rating (Figure 1a). Notably, the discrepancy between LOI and UL 94 results indicates that gas-phase flame inhibition alone is insufficient to determine fire performance. Instead, the UL 94 behavior is strongly governed by condensed-phase processes, particularly melt-dripping resistance and the rapid formation of a stable and continuous char layer [49]. Therefore, the divergence in UL 94 rating, despite similar LOI values, suggests that matrix chain architecture plays a critical role in regulating condensed-phase flame-retardant behavior [50,51].

Thermogravimetric analysis (TGA) was conducted to evaluate the thermal stability of the PP/IFR composites, and the results are presented in Figure 1b and Table S2. After the introduction of IFR, all composites exhibit a typical two-stage degradation profile under nitrogen. The first stage (T_max1_, 440–470 °C) corresponds to the decomposition of the flame retardant and the catalyzed dehydration and crosslinking of the polymer matrix. Notably, PP-3 shows a lower onset decomposition temperature (T_5%_ = 343 °C) and first maximum degradation temperature (T_max1_ = 440 °C) compared to PP-1 and PP-2, suggesting that interactions between the matrix and the IFR system may occur at an earlier stage. The second stage (T_max2_) is mainly associated with further crosslinking and stabilization of the char layer, with PP-1 exhibiting the highest char residue (9.93 wt%). These results indicate that, under identical flame-retardant formulations, chain architecture influences char-forming behavior. Specifically, higher chain regularity tends to favor increased char yield, whereas bPP promotes earlier-stage degradation and char formation.

The melt flow index (MFI) results (Figure 1d) provide preliminary insight into melt flow characteristics. The incorporation of IFR leads to an increase in MFI for all composites, indicating that the flame retardant disrupts molecular chain entanglements and induces a plasticizing effect. Among them, PP-2 exhibits the most pronounced increase in MFI, corresponding to the highest melt fluidity, whereas PP-3 shows the smallest increase. These differences in MFI suggest variations in melt resistance behavior under heat. The relatively lower fluidity of PP-3 may contribute to its improved resistance to melt deformation and dripping during combustion, which is consistent with its superior UL 94 performance.

To further elucidate the melt behavior, dynamic rheological measurements were performed (Figure 1e1–e4). All samples exhibit typical shear-thinning behavior and viscoelastic characteristics [45,52,53]. The low-frequency region reflects the material response over long timescales and is closely related to thermal softening and resistance to structural collapse. Across the entire frequency range, PP-3 displays higher storage modulus (G′) and complex viscosity (|η*|) than PP-1 and PP-2, indicating a stronger elastic response and greater resistance to flow deformation, which is important for maintaining structural integrity during combustion. Meanwhile, PP-3 shows a lower loss factor (tan δ), further suggesting a more pronounced elastic character. In contrast, PP-2 exhibits lower G′ and |η*| but higher tan δ, indicating a more fluid-like behavior with increased viscous dissipation and enhanced flowability. The rheological behavior of PP-1 lies between these two extremes.

Taken together, the superior UL 94 performance of PP-3 can be correlated with its enhanced melt resistance and viscoelastic stability, which help suppress melt dripping and support the formation of a stable intumescent char layer. In contrast, the higher fluidity of PP-2 hinders the development of a coherent protective structure during combustion.

**Figure 2:**
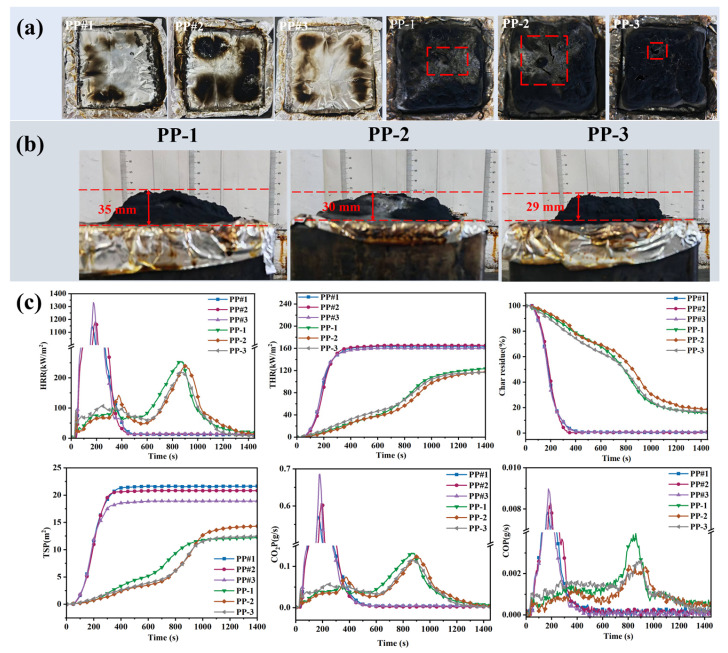
(**a**,**b**) Physical photos; (**c**) the CONE results obtained at 35 kW/m^2^.

To simulate realistic fire scenarios, cone calorimetry was conducted. The corresponding curves of heat release rate (HRR), total heat release (THR), char residue, total smoke production (TSP), CO_2_ production (CO_2_P), and CO production (COP) are presented in Figure 2c, with key parameters summarized in Table 2. Neat PP exhibits typical characteristics of intense combustion. Among the matrices, PP-2 shows the longest time to ignition (TTI) and the lowest fire growth tendency, indicating relatively slower degradation behavior. In contrast, which is likely attributed to its well-defined microphase-separated structure (hard-soft segments). The numerous interfaces in this structure act as preferential sites for initiating thermal decomposition, leading to rapid volatile release.

Upon incorporation of the IFR system, the peak heat release rate (pHRR) of all flame-retardant composites decreases by more than 80% compared to neat PP, accompanied by a substantial delay in the time to peak heat release rate (TPHRR), indicating a more gradual and retarded combustion process [54]. The fire growth rate index (FIGRA = pHRR/TPHRR) and fire performance index (FPI = TTI/pHRR) respectively reflect the rate of fire spread and the propensity of flashover after ignition [55,56,57]. Notably, PP-3 exhibits the lowest pHRR, THR, and TSP among the composites, along with a relatively high FPI, indicating superior fire safety performance [58]. This behavior may be related to the multiphase “hard–soft segment” structure of the block copolymer matrix, which could facilitate the redistribution of thermal stress and promote the formation of a more continuous intumescent char layer during the early stage of combustion [59]. In addition, the reduced total smoke release (TSR) implies a modified combustion pathway, potentially associated with improved barrier effects in the condensed phase.

In contrast, PP-2 shows a distinct behavior. Despite failing the UL 94 test at 1.6 mm, it exhibits the longest TPHRR (905 s) and the highest char residue (18.0 wt%) in cone calorimetry. This discrepancy suggests that, although PP-2 is capable of forming a relatively stable char layer at later stages, the initial formation rate or structural integrity of the char may be insufficient to effectively suppress flame propagation in the early stage. PP-1 generally shows intermediate performance; however, its relatively high peak mass loss rate (pMLR, 0.321 g/s) suggests that the formed char layer may be less resistant to structural disruption during combustion, leading to reduced stability of the protective barrier.

Overall, the cone calorimetry results demonstrate that PP chain architecture actively influences intumescent flame-retardant performance by regulating combustion behavior across different stages. Specifically, PP-3 favors rapid flame suppression and early-stage barrier formation, PP-2 contributes to higher residual char and prolonged thermal shielding at later stages, while PP-1 exhibits a balanced but less optimized performance. These results further support that flame retardancy in IFR systems is governed by distinct structural factors related to melt behavior and char evolution.

### 3.2. Residue Analysis

Digital photographs of the char residues after cone calorimetry (Figure 2a,b) provide direct visual evidence of the differences in char morphology. PP-1 forms the highest intumescent char layer; however, evident surface cracks are observed. In contrast, PP-2 exhibits a relatively low char height with a fragmented and discontinuous structure. Notably, PP-3 develops a well-defined and intact char layer with uniform expansion. This macroscopic trend is consistent with the UL 94 results, suggesting that the stability of char formation, which is closely related to melt behavior during combustion, plays a critical role in determining the integrity of the final char layer.

**Figure 3:**
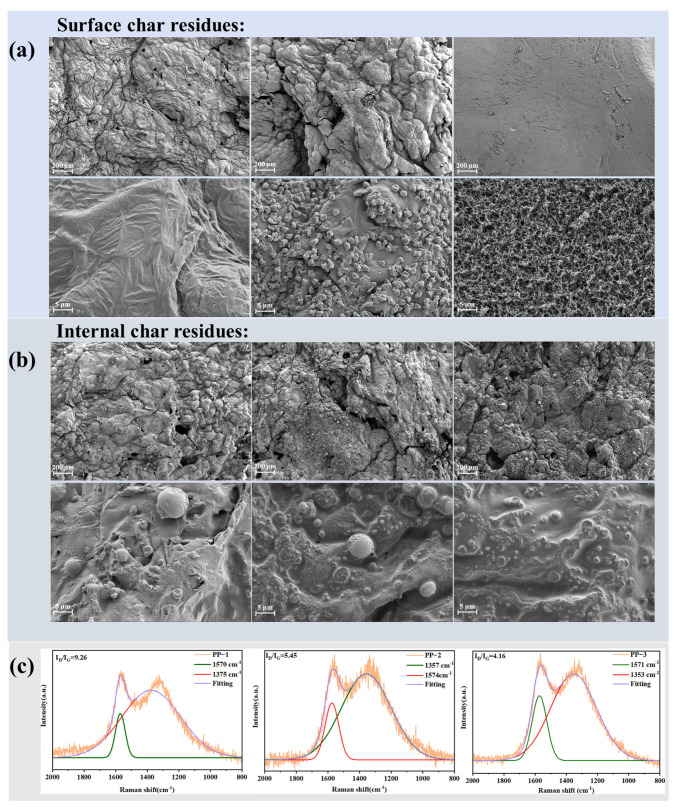
(**a**) SEM images of surface char residues; (**b**) SEM images of internal char residues; and (**c**) Raman spectroscopy of the carbon residue.

Scanning electron microscopy (SEM) was employed to further investigate the outer and inner morphologies of the char residues (Figure 3a,b). At low magnification (200 μm), the char layer of PP-1 exhibits radial cracking, indicating that the melt structure may not fully accommodate the stresses generated during rapid expansion. The PP-2 char displays a lamellar stacked structure with pronounced gaps and delamination between layers, suggesting poor structural continuity. In contrast, PP-3 forms a continuous three-dimensional porous skeleton with pore walls. At higher magnification (5 μm), PP-1 shows a wrinkled morphology, while PP-2 exhibits numerous fractured protrusions on the surface. By comparison, PP-3 presents a well-developed and interconnected carbonaceous framework. SEM observations of the inner structure further reveal that PP-3 forms a structure with smaller and more uniformly distributed pores. A well-developed char layer with higher integrity and continuity correlates with reduced heat and mass transfer in the condensed phase, a behavior reported in previous IFR-PP systems where optimized intumescent structures significantly enhanced char strength [59,60]. Conversely, PP-1 and PP-2 contain pores of varying sizes, which may compromise the overall thermal insulation and sealing performance of the char layer.

Energy-dispersive spectroscopy (EDS) elemental mapping (Table S3) reveals distinct differences in element migration and distribution. In PP-1 and PP-2, a concentration gradient of phosphorus is observed from the surface to the interior, indicating migration of flame-retardant species during char formation. In contrast, PP-3 exhibits a relatively higher carbon content at the surface, while phosphorus and nitrogen are more uniformly distributed throughout the char layer. This more homogeneous distribution is likely associated with improved dispersion and interfacial compatibility of the flame-retardant components within the PP-3 matrix, which is consistent with the more integrated char structure observed in SEM.

Raman spectroscopy (Figure 3c) was employed to evaluate the structural ordering of the char. Typical carbon materials exhibit a D band (~1350 cm^−1^), associated with disordered structures, and a G band (~1580 cm^−1^), corresponding to graphitic structures [61]. A lower intensity ratio (I_D_/I_G_) indicates a higher degree of graphitization of the char. The results show that PP-3 exhibits the lowest I_D_/I_G_ value among the composites, suggesting a relatively higher degree of graphitic organization. Such a more ordered carbon structure is generally associated with improved thermal stability and barrier properties, which may contribute to the reduced heat release and smoke production observed in cone calorimetry. In contrast, the higher I_D_/I_G_ value of PP-1 indicates a more disordered char structure, consistent with its higher mass loss rate.

Overall, the char analysis suggests that chain architecture influences the formation, structure, and composition of the intumescent char layer through differences in melt behavior and phase characteristics. In particular, the bPP-based system tends to form a more continuous, compositionally uniform, and structurally ordered char layer, whereas the rPP-based system exhibits limitations in early-stage structural integrity despite its relatively high char yield. These results further support that flame retardancy in IFR systems is governed by multiple structural factors rather than a single controlling mechanism.

### 3.3. Gas-Phase Action Analysis

**Figure 4:**
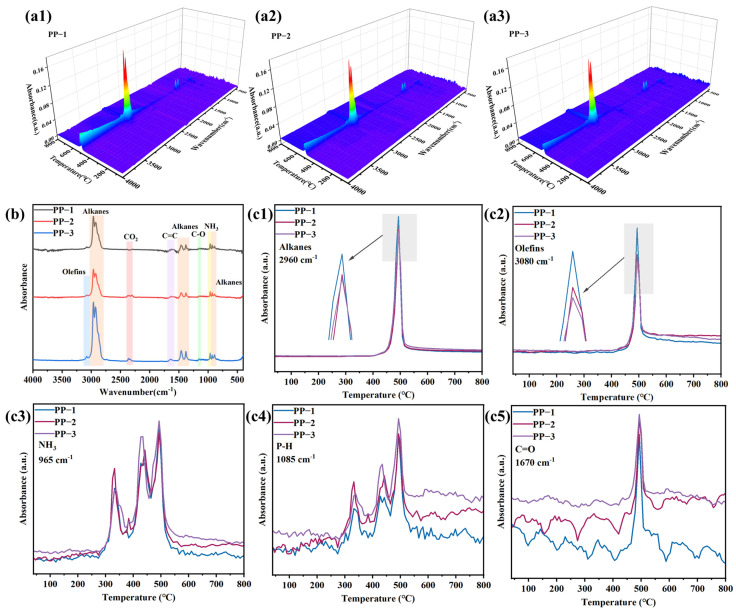
FT-IR spectra of gaseous pyrolysis products for (**a1**) PP-1, (**a2**) PP-2 and (**a3**) PP-3; characteristic evolutionary gas spectra of flame retardant composites: (**b**); characteristic peaks of the gas of interest with temperature: (**c1**–**c5**).

Thermogravimetric–infrared (TG–FTIR) analysis was employed to investigate the thermal decomposition products of the three composites (Figure 4) [62]. Colors from blue to red represent low to high absorbance values, respectively (Figure 4(a1–a3)). Under a nitrogen atmosphere, all samples exhibit characteristic signals corresponding to alkanes, alkenes, NH_3_, phosphorus-containing species, and carbonyl compounds. The release behavior of combustible volatiles is an important indicator of fire hazard. At the maximum mass loss stage (Figure 4b), the PP-3 composite shows the strongest alkane signal, followed by PP-1 and PP-2. However, further analysis of alkane (Figure 4(c1)) and alkene (Figure 4(c2)) evolution indicates that PP-1 releases the largest overall amount of combustible hydrocarbons. This observation is consistent with its relatively higher heat release in cone calorimetry, suggesting that degradation-derived volatiles from PP-1 are more likely to participate in gas-phase combustion [61]. Although PP-3 exhibits relatively strong alkane evolution at specific stages, its multiphase “hard–soft segment” structure may lead to a more complex degradation pathway. Combined with its more developed char structure and higher degree of graphitic ordering, it is likely that part of the volatile fragments are constrained by the evolving char layer, which may reduce their effective release into the flame zone.

The evolution of NH_3_ exhibits a three-stage profile (Figure 4(c3)). PP-2 shows the highest NH_3_ release in the initial stage, whereas PP-3 demonstrates sustained evolution in the subsequent stages, indicating a more continuous decomposition process of the flame-retardant system. The intensity of phosphorus-containing species (P–H-related compounds) follows the order PP-3 > PP-2 > PP-1 (Figure 4(c4)), suggesting that PP-3 generates a greater amount of phosphorus-containing species at elevated temperatures. These species are known to promote char formation in the condensed phase and may also contribute to flame inhibition in the gas phase. In addition, PP-3 exhibits relatively strong carbonyl signals (Figure 4(c5)), indicating enhanced chain scission and the formation of oxygen-containing structures under the catalytic effect of the flame retardant.

Overall, TG–FTIR analysis indicates that PP chain architecture influences the composition and evolution behavior of gaseous pyrolysis products. In particular, the bPP-based system shows a tendency toward more sustained release of phosphorus-containing species and non-flammable gases, which may contribute to both condensed-phase char development and gas-phase flame inhibition. These results further support that flame-retardant performance in IFR systems is governed by multiple interacting factors rather than a single dominant pathway.

### 3.4. Mechanical Properties

**Figure 5:**
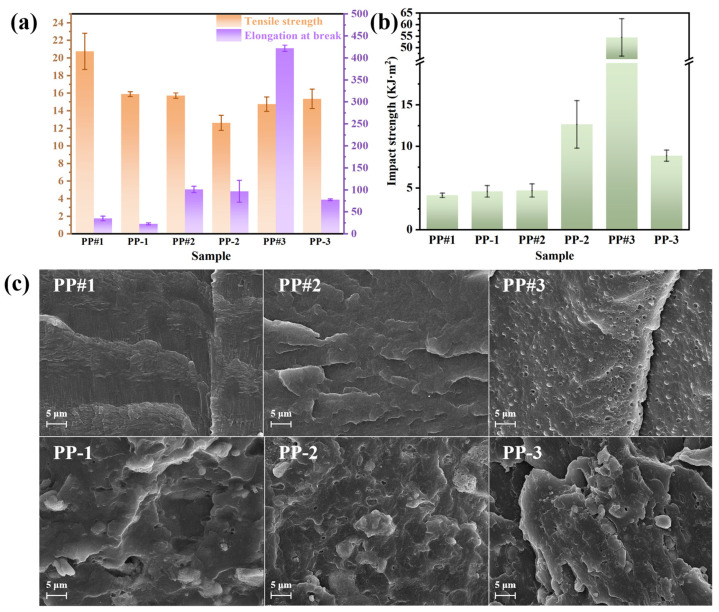
(**a**,**b**) Mechanical properties of the composites; (**c**) SEM images of the cross-section of the samples.

In contrast to flame-retardant performance, which is strongly influenced by melt rheological behavior and char evolution, the mechanical properties of the composites are primarily governed by interfacial interactions between the polymer matrix and the flame-retardant fillers [63]. The mechanical properties of neat PP (Figure 5 and Table S4) are strongly dependent on chain architecture. The block copolymer PP (PP#3), owing to the presence of a dispersed rubber phase, exhibits exceptionally high toughness and impact strength, while the random copolymer PP (PP#2) shows a higher elongation at break compared to the homopolymer PP (PP#1). After incorporation of IFR, the tensile strength of all composites decreases. However, the elongation at break of the PP-2 composite (96.8%) remains nearly unchanged compared to its neat counterpart (101.0%), whereas those of PP-1 and PP-3 decrease significantly to 22.9% and 77.6%, respectively.

Scanning electron microscopy (SEM) of the cryo-fractured surfaces (Figure 5c) provides further insight into the interfacial characteristics. In the PP-1 composite, flame-retardant particles are clearly exposed on the fracture surface, indicating weak interfacial adhesion. Such interfacial features are not favorable for stress transfer, which may contribute to the observed low elongation at break. In contrast, the PP-2 composite exhibits flame-retardant particles that are more uniformly embedded within the matrix, suggesting improved interfacial compatibility. This type of interface is beneficial for stress transfer and energy dissipation under loading, which is consistent with its relatively high ductility and improved impact strength (up to 12.6 kJ/m^2^). The mechanical performance of PP-3 reflects the sensitivity of multiphase toughening mechanisms to filler incorporation. Neat PP#3 achieves an exceptionally high impact strength of 54.5 kJ/m^2^ due to its sea–island rubber phase structure. However, after incorporation of IFR, the PP-3 composite shows a sharp reduction in impact strength to 8.9 kJ/m^2^. This change is likely associated with the disruption of the continuity of the rubber phase and the introduction of microstructural heterogeneities by the filler particles, which may act as stress concentration sites under impact loading [64,65]. Compared with the enhancement observed in PP-2, this result highlights the importance of interfacial compatibility and phase structure in determining the mechanical performance of block copolymer systems. In particular, the presence of incompatible fillers may interfere with the integrity of the multiphase morphology responsible for toughening. The results indicate that while the molecular architecture of PP significantly influences flame retardancy, the observed reductions in tensile and impact properties remain within acceptable ranges for typical applications, suggesting that the composites retain sufficient mechanical performance for practical use.

### 3.5. Crystallization Behavior

**Figure 6:**
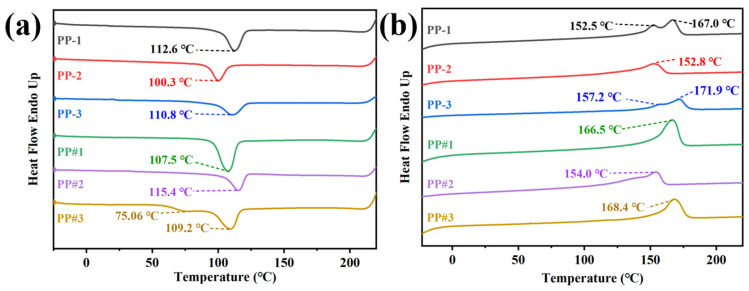
DSC curve of PP/IFR samples. (**a**) Cooling curve; (**b**) heating curve.

Differential scanning calorimetry (DSC) analysis (Table S5 and Figure 6) reveals that the thermal behavior of the neat resins reflects their intrinsic structural characteristics [29,66]. Homopolymer PP (PP#1) exhibits a relatively high crystallinity (61.0%), indicative of its regular chain structure. In contrast, random copolymer PP (PP#2) shows the lowest melting temperature and crystallinity, suggesting reduced chain regularity and a higher fraction of amorphous regions with enhanced segmental mobility. Block copolymer PP (PP#3) displays two distinct crystallization peaks, corresponding to the independent crystallization of the rigid segments and the dispersed rubber phase.

After incorporation of IFR, noticeable changes in crystallization behavior are observed. The crystallinity of the PP-2 composite decreases significantly (from 26.3% to 11.5%) (Table S5), indicating an increased contribution from the amorphous phase. Such a structure is more favorable for accommodating filler particles and may facilitate interfacial interactions through physical entanglement, which is consistent with the improved encapsulation observed in cryo-fractured SEM images. For PP-3, the melting behavior evolves from a single peak to two distinct peaks at 157.2 °C and 171.9 °C (Figure 6b), indicating the independent crystallization of the rigid segments and the dispersed rubber phase. This phenomenon reflects phase interactions within the block copolymer matrix and explains its influence on composite properties. Meanwhile, the characteristic low-temperature crystallization peak of the rubber phase (75.1 °C) disappears. This change suggests that the incorporation of IFR interferes with the crystallization of the rubber phase (Figure 6a), while possibly promoting the formation of more thermally stable crystals in the rigid segments. In the PP-1 composite, a shoulder peak corresponding to β-crystals appears at 152.5 °C (Figure 6b), indicating a heterogeneous nucleation effect induced by the flame-retardant additives. A similar β-crystal shoulder peak is also observed in the PP-3 composite, suggesting that the block copolymer matrix responds similarly to IFR-induced heterogeneous nucleation. Similar effects of fillers on the crystallization behavior of block copolymers have been reported, where filler-induced heterogeneous nucleation alters the thermal transitions of the soft segments [67,68].

Overall, the results indicate that PP chain architecture influences crystallization behavior and phase structure, which in turn affect the interaction between the polymer matrix and rigid fillers. These differences in crystallization have practical implications: for example, the higher amorphous content in PP-2 provides a more flexible matrix beneficial for maintaining ductility and ease of processing, whereas the multiphase structure of PP-3 may enhance dimensional stability but requires careful control during molding. In particular, the higher amorphous content in the random copolymer system provides a more adaptable matrix environment, which is beneficial for maintaining ductility, whereas the multiphase structure of the block copolymer is more sensitive to structural perturbations induced by filler incorporation. These findings are consistent with the observed differences in mechanical performance.

## 4. Conclusions

This work systematically compares polypropylene matrices with three distinct chain architectures—homopolymer, random copolymer, and block copolymer—under an identical intumescent flame-retardant (IFR) system, and demonstrates that polymer chain architecture plays a critical role in determining composite performance. The results show that PP chain structure influences material properties through two relatively independent pathways. Under the same flame-retardant formulation, flame retardancy is closely associated with the melt rheological behavior of the matrix, which governs melt deformation and dripping during combustion. In this context, the block copolymer PP, characterized by enhanced melt strength, which exhibits relatively higher melt viscosity and elasticity according to rheological analysis, shows improved resistance to melt dripping and enhanced flame-retardant performance. In contrast, mechanical properties, particularly toughness, are more strongly related to interfacial compatibility between the polymer matrix and the flame-retardant fillers. The random copolymer PP, owing to its higher amorphous content, provides a more adaptable matrix environment, which facilitates interfacial interactions and enables the retention of ductility along with improved impact resistance. Conversely, the multiphase structure of block copolymer PP is more sensitive to filler incorporation, leading to disruption of its toughening morphology and a reduction in impact performance. The homopolymer PP exhibits intermediate behavior between these two systems.

Overall, this study differentiates the governing factors of flame retardancy and mechanical performance in IFR polypropylene systems, highlighting the distinct roles of melt rheology and interfacial interactions. These findings provide a useful framework for the rational design of high-performance flame-retardant polymers by tailoring polymer chain architecture, rather than relying solely on the optimization of flame-retardant additives. Future work will focus on further optimization of the PP/IFR composite formulation to maximize flame retardancy while maintaining mechanical performance, as well as industrial-scale testing to assess processing and long-term stability.

## Figures and Tables

**Table 1:** The formulas (wt.%) of neat PP and the PP/IFR composites.

Sample	hPP	rPP	bPP	PAPP	APP	CF
PP#1	99.7	0	0	0	0	0
PP#2	0	99.7	0	0	0	0
PP#3	0	0	99.7	0	0	0
PP-1	74.7	0	0	14.2	7.1	3.7
PP-2	0	74.7	0	14.2	7.1	3.7
PP-3	0	0	74.7	14.2	7.1	3.7

Note: all compositions include 0.1 wt% Irganox 1010 and 0.2 wt% Irgafos 168. PP#1(hPP), PP#2(bPP), and PP#3(rPP) are neat PP, while PP-1, PP-2, and PP-3 are the corresponding PP/IFR composites.

**Table 2:** Typical parameters of PP/IFR composites in cone calorimeter test.

Sample	PP#1	PP#2	PP#3	PP-1	PP-2	PP-3
Time to ignition (TTI) (s)	33	44	23	29	32	24
Peak heat release rate (pHRR) (kW/m^2^)	1148	1182	1332	255	240	220
Time to pHRR (TPHRR) (s)	165	190	175	875	905	865
Total heat release (THR) (MJ/m^2^)	164	160	160	125	119	117
Total smoke release (TSR) (m^2^)	2450	2325	2139	1975	1626	1428
Peak effective heat of combustion (pEHC) (MJ/kg)	79.59	77.27	79.07	79.68	79.72	78.00
Peak mass loss rate (pMLR) (g/s)	0.188	0.173	0.185	0.321	0.128	0.067
Char residue (%)	0.393	0.859	0.376	14.73	18.00	16.75
Fire performance index (FPI) ((m^2^·s)/kW)	0.0287	0.0372	0.0173	0.1139	0.1331	0.1091
Fire growth rate index (FIGRA) (kW/(m^2^·s))	6.957	6.223	7.610	0.281	0.266	0.254

## Data Availability

The original contributions presented in this study are included in the article and Supplementary Material. Further inquiries can be directed to the corresponding author.

## Supplementary Materials

The following supporting information can be downloaded at: https://www.mdpi.com/article/10.3390/polym18111356/s1, Table S1: The LOI and UL 94 rating of neat PP and PP/IFR composites; Table S2: TG and DTG data of PP/IFR composites under N2 atmosphere; Table S3: Elemental composition (in atomic percentage, at%) of the cone calorimeter test residues for PP-1, PP-2, and PP-3 composites, obtained from energy-dispersive X-ray spectroscopy (EDS) analysis on the surface and internal cross-sections; Table S4: Mechanical property data of neat PP and PP/IFR samples; Table S5: DSC and crystallization data of PP and its composites.
