# Supplementary material for: Dominant Role of Polypropylene Chain Architecture in Differentiating Flame Retardancy and Mechanical Performance
Source: Polymers (Basel). 2026 May 29;18(11):1356. doi: 10.3390/polym18111356 (PMC13259468; doi:10.3390/polym18111356)
Supplement: Supplementary file 1 [file polymers-18-01356-s001.zip › polymers-4319931-supplementary.pdf]

# Supporting Information

## Dominant Role of Polypropylene Chain Architecture in Differentiating Flame Retardancy and Mechanical Performance

Shu Yin[a, b], Menghan Guo [a, b], Hao Wang[a, b], Lin Wang[a, b], Xiangmei Li[a, b*], Jiyu He[a, b]

a School of Material Science and Engineering, Beijing Institute of Technology, Beijing 10081, China

b National Engineering Research Center of Flame Retardant Materials, Beijing Institute of Technology, Beijing 10081, China

*Corresponding Author:
Xiangmei Li. E-mail: bjlglxm@bit.edu.cn

Table S1. The LOI and UL-94 rating of neat PP and PP/IFR composites

| Sample | UL 94,3.2 mm bar | | | | UL 94,1.6 mm bar | | | | LOI（%） |
|---|---|---|---|---|---|---|---|---|---|
| | $t_1$/s | $t_2$/s | dripping | rating | $t_1$/s | $t_2$/s | dripping | rating | |
| PP#1 | - | - | Y[a] | NR | - | - | Y | NR | 17.5 |
| PP#2 | - | - | Y | NR | - | - | Y | NR | 17.5 |
| PP#3 | - | - | Y | NR | - | - | Y | NR | 18.0 |
| PP-1 | 1 | 2 | N[b] | V-0 | 1 | 4 | N | V-0 | 31.5 |
| PP-2 | 1 | 2 | N | V-0 | 9 | - | Y | NR | 31.5 |
| PP-3 | 1 | 1 | N | V-0 | 1 | 5 | N | V-0 | 32.5 |

Note:

PP#1, PP#2, and PP#3 are neat PP, while PP-1, PP-2, and PP-3 are the corresponding PP/IFR composites.
- :- represents the specimen burned up to the holding clamp.

a : Y represents molten droplets production, which ignites the absorbent cotton below.

b : N represents no molten droplets falling.

Table S2. TG and DTG data of PP/IFR composites under $N_2$ atmosphere.

| Sample | $T_{5\%}$(°C) | $T_{max1}$(°C) | $T_{max2}$(°C) | Char yield at 800°C（wt%） |
|---|---|---|---|---|
| PP#1 | 418 | 463 | - | 0.16 |
| PP#2 | 416 | 457 | - | 0.19 |
| PP#3 | 427 | 456 | - | 1.30 |
| PP-1 | 366 | 466 | 531 | 9.93 |
| PP-2 | 369 | 470 | 526 | 6.30 |
| PP-3 | 343 | 440 | 574 | 6.37 |

Table S3. Elemental composition (in atomic percentage, at%) of the cone calorimeter test residues for PP-1, PP-2, and PP-3 composites, obtained from energy-dispersive X-ray spectroscopy (EDS) analysis on the surface and internal cross-sections.

| Sample | PP-1 | PP-1 | PP-2 | PP-2 | PP-3 | PP-3 |
|---|---|---|---|---|---|---|

| | Surface | Internal | Surface | Internal | Surface | Internal |
|---|---|---|---|---|---|---|
| N(at%) | 7.37 | 6.37 | 7.21 | 6.37 | 1.55 | 5.21 |
| P(at%) | 5.43 | 10.24 | 8.07 | 10.24 | 4.34 | 10.48 |
| C(at%) | 67.26 | 40.36 | 54.88 | 49.36 | 75.48 | 46.64 |
| O(at%) | 19.94 | 34.03 | 29.84 | 34.03 | 18.63 | 37.68 |

Table S4. Mechanical property data of neat PP and PP/IFR samples

| Sample | Tensile strength （MPa） | Elongation at break （%） | Izod（kJ/㎡） |
|---|---|---|---|
| PP#1 | 20.8±0.2 | 35.0±5.3 | 4.1±0.3 |
| PP-1 | 15.9±0.3 | 22.9±2.3 | 4.6±0.7 |
| PP#2 | 15.7±0.3 | 101.0±7.2 | 4.7±0.8 |
| PP-2 | 12.6±0.9 | 96.8±24.8 | 12.6±2.9 |
| PP#3 | 14.8±0.8 | 422.1±7.0 | 54.5±8.1 |
| PP-3 | 15.4±1.1 | 77.6±2.0 | 8.9±0.7 |

Table S5. DSC and crystallization data of PP and its composites.

| Sample | $T_m$(°C) | $T_c$(°C) | $\Delta H_m$ (J/g) | $\Delta H_c$ (J/g) | Crystallinity(%) | ΔCrystallinity(%) |
|---|---|---|---|---|---|---|
| PP#1 | 166.5 | 107.5 | 127.5 | 122.1 | 61.0 | - |
| PP#2 | 154.0 | 115.4 | 53.9 | 72.6 | 26.3 | - |
| PP#3 | 168.4 | 75.1/109.2 | 68.0 | 120.6 | 32.6 | - |
| PP-1 | 152.5/167.0 | 112.6 | 58.7 | 93.6 | 28.1 | 23.3 |
| PP-2 | 152.8 | 100.3 | 24.0 | 67.7 | 11.5 | 56.2 |
| PP-3 | 157.2/171.9 | 110.8 | 53.3 | 49.0 | 25.5 | 21.7 |
